# Myocardial haemorrhage after acute reperfused ST-elevation myocardial infarction evolves progressively and contributes to the early bimodal pattern in T2-relaxation time: advanced imaging and clinical significance

**DOI:** 10.1186/1532-429X-18-S1-P231

**Published:** 2016-01-27

**Authors:** David Carrick, Caroline Haig, Nadeem Ahmed, Samuli M Rauhalammi, Guillaume Clerfond, jaclyn carberry, Ify Mordi, Margaret McEntegart, Mark Petrie, Hany Eteiba, Stuart Hood, Stuart Watkins, Mitchell Lindsay, Ahmed Mahrous, Paul Welsh, Naveed Sattar, Ian Ford, Keith G Oldroyd, Aleksandra Radjenovic, Colin Berry

**Affiliations:** 1 grid.413157.50000000405902070Golden Jubilee National Hospital, Clydebank, United Kingdom; 2 Institute of Cardiovascular and Medical Sciences, BHF Glasgow Cardiovascular Research Centre, Glasgow, United Kingdom; 3 grid.8756.c000000012193314XRobertson Center for Biostatistics, University of Glasgow, Glasgow, United Kingdom

## Background

The time-course and relationships of myocardial haemorrhage and oedema in survivors of acute ST-elevation myocardial infarction (STEMI) are uncertain.

## Methods

30 STEMI patients (mean age 54 years; 25(83%) male) treated by primary percutaneous coronary intervention underwent serial cardiac magnetic resonance imaging: 4 - 12 hours, 3 days, 10 days and 7 months post-reperfusion. Native T2 and T2* were measured in regions-of-interest in remote and injured myocardium. Myocardial haemorrhage was taken to represent a hypointense infarct core with a T2* value <20 ms. Public registration: NCT02072850.

## Results

Myocardial haemorrhage occurred in 7(23%), 13(43%), 11(33%), and 4(13%) patients at 4 - 12 hours, 3 days, 10 days and 7 months, consistent with a unimodal pattern. The corresponding amounts of myocardial haemorrhage (% LV mass) during the first 10 days post-MI were (median, IQR): 2.7(0.0, 5.6), 7.0(4.9, 7.5), 4.1(2.6, 5.5); p < 0.001). Myocardial oedema (% LV mass) had a unimodal evolution in all patients (p=0.001). In patients without hemorrhage, infarct zone T2 values (ms) increased progressively during the first 10 days (62.1(2.9), 64.4(4.9), 65.9(5.3) (p < 0.001). Alternatively, in patients with myocardial haemorrhage, infarct zone T2 was reduced at day 3 (51.8 (4.6) ms) (p < 0.001), depicting a bimodal pattern.

LV end-diastolic volume increased from baseline to 7 months in patients with myocardial haemorrhage (p=0.001), but not in patients without haemorrhage (p=0.377).

## Conclusions

The temporal evolutions of myocardial haemorrhage and oedema are unimodal, whereas infarct zone T2 (ms) has a bimodal pattern in haemorrhagic infarction. Myocardial haemorrhage is prognostically important. Further studies are warranted.

**Figure 1:**
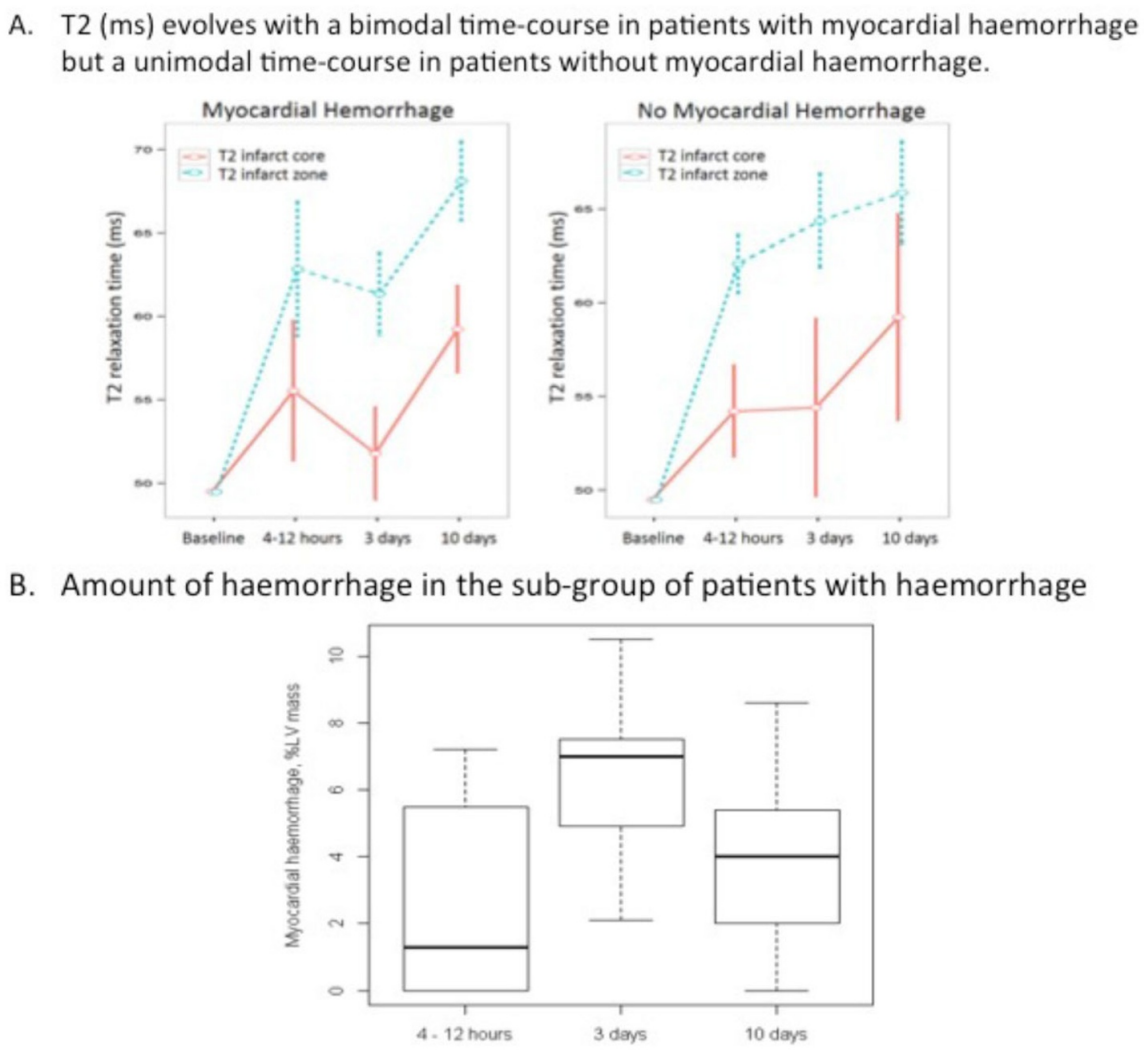
Figure 1

